# Transfer of improved movement technique after receiving verbal external focus and video instruction

**DOI:** 10.1007/s00167-017-4671-y

**Published:** 2017-08-10

**Authors:** Anne Benjaminse, Wouter Welling, Bert Otten, Alli Gokeler

**Affiliations:** 1 Center for Human Movement Science, University Medical Center Groningen, University of Groningen, Antonius Deusinglaan 1, 9713 AV Groningen, The Netherlands; 2 School of Sport Studies, Hanze University Groningen, Zernikeplein 17, 9747 AS Groningen, The Netherlands; 3 Medisch Centrum Zuid, Sportlaan 2-1, 9728 PH Groningen, The Netherlands

**Keywords:** Motor learning, Movement technique, Transfer, Self-controlled feedback

## Abstract

**Purpose:**

It is unknown how movement patterns that are learned carry over to the field. The objective was to determine whether training during a jump-landing task would transfer to lower extremity kinematics and kinetics during sidestep cutting.

**Methods:**

Forty healthy athletes were assigned to the verbal internal focus (IF, *n* = 10), verbal external focus (EF, *n* = 10), video (VI, *n* = 10) or control (CTRL, *n* = 10) group. A jump-landing task was performed as baseline followed by training blocks (TR1 and TR2) and a post-test. Group-specific instructions were given in TR1 and TR2. In addition, participants in the IF, EF and VI groups were free to ask for feedback after every jump during TR1 and TR2. Retention was tested after 1 week. Transfer of learned skill was determined by having participants perform a 45° unanticipated sidestep cutting task. 3D hip, knee and ankle kinematics and kinetics were the main outcome measures.

**Results:**

During sidestep cutting, the VI group showed greater hip flexion ROM compared to the EF and IF groups (*p* < 0.001). The EF (*p* < 0.036) and VI (*p* < 0.004) groups had greater knee flexion ROM compared to the IF group.

**Conclusions:**

Improved jump-landing technique carried over to sidestep cutting when stimulating an external attentional focus combined with self-controlled feedback. Transfer to more sport-specific skills may demonstrate potential to reduce injuries on the field. Clinicians and practitioners are encouraged to apply instructions that stimulate an external focus of attention, of which visual instructions seem to be very powerful.

**Level of evidence:**

II.

## Introduction

As anterior cruciate ligament (ACL) injuries continue to rise [1], there is a need for improvement in current injury prevention programmes. A common denominator of these programmes is that instructions addressing desired movement form are mostly given with an internal focus (IF) of attention [3]. An IF of attention means that the athlete focusses on the body and movement, whereas with an external focus (EF) of attention the athlete is focused on the movement effect [28]. It has been shown that verbal EF and visual instructions stimulate implicit motor learning and, with this, enhance movement technique over time (i.e. retention) [3, 7, 13, 16, 26]. This indicates that the effects of verbal EF and visual instructions have the potential to become relatively permanent, rather than temporary [21].

Besides retention, the transfer of a learned motor skill to a sport-specific situation on the field is important, as it gives an indication on how the athlete is able to use a wide spectrum of skills in different situations. However, research has been sparse [7]. The most optimal way to enhance transfer of sport-specific skills is still not known. This is, however, crucial for prevention as most of the actual injuries happen on the field [1].

Giving the athlete some autonomy over a practice session may enhance motor skill learning in comparison with prescribed training schedules [2, 28]. Self-controlled learning increases motivation and therefore enhances the efforts invested in practice [28]. In addition, feedback emphasising successful trials benefits learning through increased perceptions of competence and self-efficacy [20]. It is not known yet how this specifically applies to motor performance and technique and its transfer in the domain of ACL injury prevention.

The primary purpose of the present study was thus to determine whether the instruction related to a jump-landing task with self-controlled feedback would transfer to lower extremity kinematics and kinetics during sidestep cutting, comparing a verbal EF, verbal IF, video (VI) and control (CTRL) group. It was hypothesised that the EF and VI groups demonstrate better movement technique (i.e. reduced load at the knee) in the transfer test, compared to the IF and CTRL groups. The secondary objective was to investigate the timing of feedback, to start exploring whether the mediating role of self-controlled feedback on transfer of learning offers an explanation on the superiority of easy-to-difficult transfer [23].

## Materials and methods

A randomised controlled trial was conducted in a controlled laboratory setting. Twenty male and twenty female participants (22.5 ± 1.6 years, 179.7 ± 0.4 cm, 74.0 ± 12.7 kg) were recruited from local sports clubs, representative of a random sample of a larger population. Enrolment, random allocation and testing were conducted by W.W. Subjects were allocated with a MATLAB 6.1 (The MathWorks Inc., Natick, MA) randomisation script to one of the four groups based on sex, age and length: IF group with verbal instructions (*n* = 10), EF group with verbal instructions (*n* = 10), VI instructions group (*n* = 10) or the CTRL group (*n* = 10) with no specific instruction. For inclusion, participants had to be: (1) ≥18 years old and (2) physically active in recreational ball team sports for a minimum of 4 h per week. Subjects were excluded if (1) they had lower extremity injury or surgery in the past 6 months or (2) they ever had a knee surgery. Prior to testing, all participants signed an informed consent form.

First, expert videos were made available in the database to provide instruction to the VI group (expert modelling). Before recording the expert jump-landing tasks, general anthropometric measures were taken from the expert athletes. They had 21 reflective markers of 14 mm in diameter placed according to the Vicon Plug-in-Gait marker set and model. In addition, trunk markers were added to the sternum, clavicle, C7, T10 and right scapula. This was followed by a static calibration. Kinematic data were collected using an eight camera motion analysis system at 200 Hz [Vicon Motion Analysis Systems Inc., Oxford, UK and Vicon Nexus software (version 1.8.3, Oxford, UK)]. Good measurement accuracy and high test and retest repeatability have been previously reported [10, 11]. Ground reaction force data were collected at 1000 Hz with two Bertec force plates (Bertec Corporation, Columbus, OH). The methods for the expert videos have been described in detail previously [26].

For included participants, same preparation procedures as with the expert participants were followed. After completing a 5-min warm-up, the participants received the general instructions and practiced the double-legged jump-landing task to familiarise themselves. The task was performed according to the protocol by Padua et al. [14]. During general task instruction, emphasis was placed on jumping as high as possible after landing from the box: “this is a jump-landing and the goal is to jump as high as possible after you have landed on the floor”. Landing technique was assessed from the jump-landing task in five sessions: pretest (five baseline trials), two training blocks (TR1 and TR2, each ten trials) and directly after the training sessions (post-test, five trials). After the pretest, group-specific instructions were given and repeated after every five trials. For the IF group: “extend your knees as rapidly as possible after the landing on the force plate”. For the EF group: “push yourself as hard as possible off the ground after landing on the force plate”. The VI group watched the contour of an expert performing the jump-landing task and were instructed to imitate the expert as good as possible. In addition, participants in the IF, EF and VI groups were free to ask for feedback after every jump in TR1 and TR2 (i.e. self-controlled feedback). This feedback consisted of their real-time landing error scoring system (LESS) score of that respective jump [14]. Subjects were only aware that a lower LESS score implied a better landing technique.

Retention was tested 1 week later, consisting of five jump-landing trials with only the general instruction provided, followed by five 45° unanticipated sidestep cutting trials as a transfer test. Full details on materials and methodology of this task can be found in previous research [3]. The study was approved by the medical ethical board of the University of Groningen (ECB/2014.1.20_1).

## Data acquisition and statistical analysis

Moments are expressed as external moments normalised to body weight. Results in degrees will be reported to one decimal case [27]. Primary outcome variables were vGRF, trunk, hip and knee sagittal joint angles and moments. In addition, frontal plane moments for the knee were collected. All variables were expressed at peak external valgus/varus moment, because this parameter has been associated with increased ACL injury risk. Range of motion (ROM) was calculated as the value at peak external valgus/varus moment minus the value at the initial contact. Moments are expressed as external moments normalised to body weight. Based on number of participants and pooled standard deviation, ESs were calculated for all comparisons using Cohen’s *d* values where 0.2 ≤ *d* ≤ 0.5, 0.5 ≤ *d* ≤ 0.8 and *d* ≥ 0.8 represent a small, moderate and large effect, respectively [4]. All frontal and sagittal jump-landing videos were independently analysed and scored (W.W. and A.B.) [26], using the LESS [15].

Customised software using MATLAB 6.1 (The MathWorks Inc., 220 Natick, MA) was written and used to compute segmental kinematics and kinetics for both legs (jump-landing) and dominant leg (sidestep). Force plate and kinetic data were filtered using a fourth-order zero-lag Butterworth low-pass filter at 10 Hz. Assumptions for normality of distribution for all variables were checked, and homogeneity of variance and sphericity were also validated for the use of analysis of variance (ANOVA). A multivariate 2 × 4 ANOVA was conducted to examine differences in sidestep cutting technique between groups (IF, EF, VI and CTRL) and sex (female and male). This was followed by post hoc comparisons (Bonferroni) with alpha level set at *α* ≤ 0.05 a priori. Additionally, the timing of requested feedback of the IF, EF and VI groups was calculated. To reach an effect size (ES) of 0.25 (medium effect ANOVA) [5], an alpha of 0.05 and a power of 0.80, 10 participants were needed per group (G*Power for Windows, Version 3.1.7.).

## Results

At baseline, no significant differences in LESS, kinematics and kinetics on the jump-landing task were found across all groups (manuscript in revision). The results of the jump-landing task show that males in the VI group and females both in the VI and EF groups significantly improved jump-landing technique (average males and females: pretest EF LESS = 3.08, retention EF LESS = 2.34, pretest VI LESS = 2.78, retention VI LESS = 1.96) [26].

**Fig. 1:**
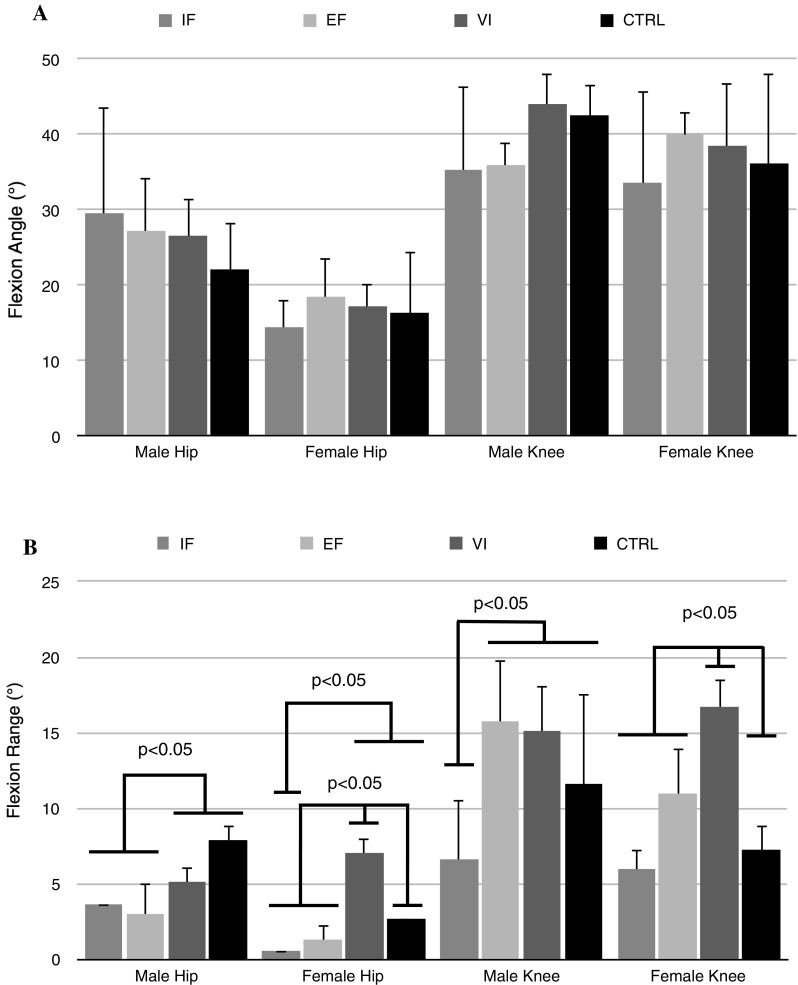
**a** Hip and knee flexion angles (expressed at peak varus/valgus moment). **b** Hip and knee flexion ranges of motion for females (expressed at peak varus/valgus moment). *IF* internal focus group, *EF* external focus group, *VI* video group, *CTRL* control group. Knee flexion is converted to positive in this figure for display purposes

Results of the transfer test are shown in Fig. 1a, b and Tables 1 and 2.

**Table 1:** Kinematic and kinetic results of the sidestep cutting task

Variable (@ peak valgus/varus moment)	Male				Female			
	IF	EF	VI	CTRL	IF	EF	VI	CTRL
Trunk flexion angle (°)	17.1 ± 6.3	11.9 ± 3.7	12.2 ± 2.3	11.0 ± 4.1	23.6 ± 5.3	21.4 ± 5.9	19.9 ± 5.0	18.0 ± 5.3
Hip flexion angle (°)	29.5 ± 13.6	27.2 ± 6.9	26.5 ± 5.4	22.2 ± 5.8	14.4 ± 3.7	18.5 ± 5.0	17.2 ± 2.9	16.4 ± 8.1
Knee flexion angle (°)	−35.3 ± 11.0	−36.0 ± 3.4	−44.1 ± 3.6	−42.6 ± 3.5	−33.5 ± 12.2	−39.9 ± 2.5	−38.5 ± 8.3	−36.1 ± 12.0
Hip flexion range (°)	3.7 ± 0.0	3.0 ± 1.5	5.1 ± 1.0	5.1 ± 1.0	0.6 ± 0.0	1.3 ± 0.7	7.0 ± 1.0	2.7 ± 0.0
Knee flexion range (°)	−6.6 ± 3.9	−15.8 ± 3.9	−15.1 ± 2.6	−11.6 ± 5.6	−6.0 ± 1.3	−11.0 ± 3.0	−16.7 ± 1.8	−7.3 ± 1.6
Knee extension (+)/flexion (−) moment (Nm/kg)	−1.60 ± 0.98	−1.36 ± 1.43	−1.79 ± 1.29	−1.75 ± 0.96	−1.06 ± 0.86	0.97 ± 0.73	−1.85 ± 0.27	−1.22 ± 0.51
Knee varus moment (Nm/kg)	0.59 ± 0.38	0.24 ± 0.69	0.72 ± 0.22	1.03 ± 0.57	0.63 ± 0.20	0.40 ± 0.26	0.71 ± 0.29	0.61 ± 0.43
vGRF (N/kg)	20.37 ± 9.64	21.78 ± 9.36	19.04 ± 5.55	18.44 ± 5.11	18.80 ± 5.72	23.10 ± 7.24	18.99 ± 5.56	17.52 ± 6.66

*IF* internal focus group, *EF* external focus group, *VI* video group, *CTRL* control group, *vGRF* vertical ground reaction force

**Table 2:** ANOVA results of within and between group analysis and effect sizes of the sidestep cutting task

Variable (@ peak valgus/varus moment)	*p* value group + ES for males	*p* value group + ES for females	*p* value sex + ES (regardless of group)	*p* value group + ES (regardless of sex)	*p* value + ES sex * group	*p* value + ES IF sex	*p* value + ES EF sex	*p* value + ES VI sex	*p* value + ES CTRL sex
Trunk flexion angle (°)	n.s.	n.s.	<0.001 ES = −1.51	n.s.	n.s.	n.s.	0.017 (ES = −1.93)	0.015 (ES = −1.98)	n.s.
Hip flexion angle (°)	n.s.	n.s.	<0.001 ES = 1.35	n.s.	n.s.	n.s.	0.050 (ES = 1.44)	0.009 (ES = 2.15)	n.s.
Knee flexion angle (°)	n.s.	n.s.	n.s.	n.s.	n.s.	n.s.	n.s.	n.s.	n.s.
Hip flexion range (°)	0.017 (EF vs. VI) ES = −1.64 < 0.001 (EF vs. CTRL) ES = −4.30 < 0.001 (IF vs. CTRL) ES = −11.20 0.002 (VI vs. CTRL) ES = −3.46	0.010 (IF vs. CTRL) ES = −2.04 <0.001 (VI vs. EF) ES = 6.50 <0.001 (VI vs. IF) ES = 8.93 <0.001 (VI vs. CTRL) ES = 6.05	<0.001 ES = 0.73	<0.001 (EF vs. VI) ES = −2.78 <0.001 (EF vs. CTRL) ES = −1.57 <0.001 (IF vs. VI) ES = −2.42 <0.001 (IF vs. CTRL) ES = −1.33	<0.001 ES = −0.03	<0.001 (ES = 3.15)	0.050 (ES = 1.45)	0.019 (ES = −1.90)	<0.001 (ES = 4.16)
Knee flexion range (°)	0.003 (EF vs. IF) ES = −2.36 0.005 (VI vs. IF) ES = −2.56	0.044 (EF vs. IF) ES = −2.15 0.007 (VI vs. EF) ES = −2.25 < 0.001 (VI vs. IF) ES = −6.73 <0.001 (VI vs. CTRL) ES = −5.45	0.044 ES = −0.51	0.036 (EF vs. IF) ES = −1.00 0.004 (VI vs. IF) ES = −1.73	n.s.	n.s.	n.s.	n.s.	n.s.
Knee extension (+)/flexion (−) moment (Nm/kg)	n.s.	n.s.	n.s.	n.s.	n.s.	n.s.	n.s.	n.s.	n.s.
Knee varus moment (Nm/kg)	n.s.	n.s.	n.s.	n.s.	n.s.	n.s.	n.s.	n.s.	n.s.
vGRF (N/kg)	n.s.	n.s.	n.s.	n.s.	n.s.	n.s.	n.s.	n.s.	n.s.

*IF* internal focus group, *EF* external focus group, *VI* video group, *CTRL* control group, *vGRF* vertical ground reaction force, *ES* effect size

For hip flexion ROM, a main effect of group showed that the VI group had greater hip flexion ROM compared to the EF and IF groups (*p* < 0.001). For males, the VI and CTRL groups showed greater hip flexion ROM compared to the IF and EF groups (*p* < 0.05). For females, the VI group showed greater hip flexion ROM compared to the EF, IF and CTRL groups (*p* < 0.05). Females in the VI group showed greater hip flexion ROM compared to the males in the VI group (*p* = 0.019).

There was a main effect of group where the EF (*p* < 0.036) and VI (*p* < 0.004) groups had greater knee flexion ROM compared to the IF group. The VI group showed greater knee flexion ROM than the EF, IF and CTRL groups for females (*p* < 0.05). Lastly, males showed greater knee flexion ROM compared to females (*p* < 0.001).

Within the EF and VI groups, females showed greater trunk flexion angles compared to males (EF *p* = 0.017, VI *p* = 0.015) and males showed greater hip flexion angles (EF *p* = 0.050, VI *p* = 0.009) compared to females.

Feedback timing during the jump-landing task in TR1 and TR2 is shown in Table 3. As a trend, participants asked for feedback typically after they performed a good jump-landing task (low or equal LESS score), this was especially the case in the EF and VI groups.

**Table 3:** Feedback timing per group per training session, after lower, equal or higher LESS score, respectively

	Lower TR1	Equal TR1	Lower and equal TR1	Higher TR1	Lower TR2	Equal TR2	Lower and equal TR2	Higher TR2
IF	9 (30%)	11 (37%)	20 (67%)	10 (33%)	8 (33%)	7 (29%)	15 (63%)	9 (41%)
EF	3 (20%)	6 (40%)	9 (60%)	6 (40%)	0 (0%)	12 (86%)	12 (86%)	2 (14%)
VI	6 (27%)	13 (59%)	19 (86%)	3 (14%)	3 (14%)	16 (73%)	19 (86%)	3 (14%)

*IF* internal focus group, *EF* external focus group, *VI* video group, *TR1* training session 1, *TR2* training session 2, *lower* request feedback after lower LESS score, *equal* request feedback after equal LESS score, *higher* request feedback after higher LESS score

## Discussion

The main finding of this study is that instructions on a simple jump-landing task did transfer to movement technique in a more sport-specific task in those who received EF verbal or VI instructions. Especially, the VI group receiving visual instruction seemed to be effective in adopting a safe sidestep cutting technique during transfer, while maintaining their performance (i.e. running speed). With this, the hypothesis was confirmed. In addition, especially participants in the EF and VI groups asked for feedback typically after they performed a relatively good jump-landing task.

Generally speaking, the males in the EF and VI groups were more effective in using the hip and knee sagittal movement to absorb energy compared to females (Fig. 1b). It is interesting to note that the females receiving VI instructions were as affective as the males. In addition, the females in the VI group landed softer compared to females in the other groups (EF, IF and CTRL). This is in contrast to a previous study where females were not as responsive to receiving visual feedback from their own trials compared to males [3]. Even though they were watching their own best trials, they still were looking at relatively “suboptimal” landing styles. Whereas in the current study, observation of a skilled (expert) model could have facilitated the development of a correct movement [18]. Hip and knee ROM during sidestep cutting was smaller though compared to others [3, 6, 9]. Maybe this is because a set completion time was required, instead of a personal percentage, creating less time to use the full potential of ROM [6, 9]. Or maybe more comprehensive instruction/feedback is necessary to enhance transfer of soft movement strategy even more [3].

Females seemed to rely more on using a trunk strategy than the males. Post hoc analysis showed that females in the EF and VI groups used greater trunk flexion angles than the males in these groups. This is in accordance with previous findings [3], where the females in the VI group increased trunk flexion angle over time. By moving the trunk forward, the distance of the vGRF to the knee becomes smaller requiring less quadriceps activity. Ultimately, the absorption of energy is dissipated over multiple joints, including the knee. This is not seen in the IF group, with only 6.0° of knee ROM, compared to 11.0° (*p* = 0.044) and 16.7° (*p* < 0.001) in the EF and VI groups, respectively.

Each group showed comparable vGRF’s, meaning the effective stiffness of the legs was the same. As there were differences in angles between groups, as explained above, this can be attributed to the direction of the vGRF and the active involvement of quadriceps, especially in the EF and VI groups showing greater knee flexion ROM. This has an effect on loading rate as these participants use more muscular activity to dissipate forces.

The EF and VI groups in general showed a more favourable movement technique during sidestep cutting transfer task compared to the IF and CTRL groups. This can be attributed to three main factors. First, external focus instructions: a focus on the movement effect (i.e. goal) promotes the utilisation of unconscious or automatic processes and, with this, enhances the production of effective and efficient movement patterns [2, 28].

Second, the participants who practiced with video instructions alternated between practicing and observing, which has been shown effective for transfer [22]. This “whole-body approach” (participants were instructed to imitate the expert on the video, without pinpointing at specific body parts) enhances being embedded in the task (embodied cognition) and appears to be an effective method to promote motor learning [2, 6].

Lastly, knowledge of results (LESS score) in the EF and VI groups was self-chosen after perceived successful trials (Table 3). Feedback after good trials plays a strong role in confirmation of competence and enhancing intrinsic motivation [20]. This enhances subsequent learning when processing feedback [8]. Confirmation of superior performance, the participants in the EF and VI groups knew that their jump-landing technique got better, is associated with higher levels of self-efficacy [12, 20, 23]. This form of self-confidence provides a buffer against stress which can explain the increased transfer with self-controlled learning [24]. It also could very well have attributed to the benefits of practicing an easier task before a more difficult one [23].

Several limitations of this study need to be addressed. First, findings of this study may be limited to this specific population of recreational athletes. The expert reference values were mostly based on female ACL injury risk factors because the literature regarding ACL injury risk factors for male athletes is scarce [25]. For future studies, it would be useful to add questionnaires on feedback mode, frequency, timing, self-efficacy and learning preferences [17]. For example, it would be interesting to examine the effect of giving athletes the choice to receive visual and/or verbal instruction. With this, we can better tailor towards individual needs, which is important in ACL injury prevention. Furthermore, the accuracy of skin-based markers in estimating joint kinematics and kinetics has been questioned [11]. Lastly, no baseline sidestep cutting data have been collected. Even though this is a common design for transfer research [7, 19, 26], it is useful for future studies to have this included to be able to examine changes for this specific task.

Suggestions for clinical and practical use are (1) to apply instructions that stimulate the use of an external focus of attention, (2) visual instructions seem to be very powerful and it is therefore suggested to add these types of instructions in ACL injury prevention programmes. It can be easily implemented in the field through the use of simple technology (tablet, smartphone) and (3) to approach ACL injury prevention from a behavioural and social–cognitive perspective. Give learners some form of autonomy to potentially enhance motivation. Future research should include investigating whether learned movement techniques remain during a practice or game.

## Conclusion

Improved movement technique carried over from a relatively easy to more difficult athletic task when receiving verbal EF and VI instruction combined with self-controlled feedback on movement form. Participants maintained performance during the transfer test, i.e. running speed. Feedback in the EF and VI groups was predominantly requested after good trials. It is suggested to allow a form of self-controlled feedback which enhances self-efficacy and autonomy and, with this, motor performance and technique. It is therefore advised to also approach ACL injury prevention from a behavioural and social–cognitive perspective. The fact that visual and/or verbal external focus instructions enhance transfer to another task underlines this to be a very powerful mode for motor learning.
